# Simultaneous changes in visual acuity, cortical population receptive field size, visual field map size, and retinal thickness in healthy human aging

**DOI:** 10.1007/s00429-021-02338-0

**Published:** 2021-07-10

**Authors:** Maria Fatima Silva, Ben M. Harvey, Lília Jorge, Nádia Canário, Fátima Machado, Mário Soares, Otília C. d’Almeida, Miguel Castelo-Branco

**Affiliations:** 1 Faculty of Medicine, Coimbra Institute for Clinical and Biomedical Research (iCBR), University of Coimbra, Coimbra, Portugal; 2 Coimbra Institute for Biomedical Imaging and Translational Research (CIBIT), University of Coimbra, Coimbra, Portugal; 3 Department of Experimental Psychology, Helmholtz Institute, Utrecht University, Heidelberglaan 1, 3584 CS Utrecht, The Netherlands; 4 Institute of Nuclear Sciences Applied to Health (ICNAS), University of Coimbra, Coimbra, Portugal; 5 CHUC, Ophthalmology Department, University of Coimbra, Coimbra, Portugal

**Keywords:** Visual cortex, Aging, Retinal thickness, Visual acuity, fMRI, Population receptive field (pRF) modeling

## Abstract

Healthy human aging is associated with a deterioration of visual acuity, retinal thinning, visual field map shrinkage and increasing population receptive field sizes. Here we ask how these changes are related to each other in a cross-sectional sample of fifty healthy adults aged 20–80 years. We hypothesized that age-related loss of macular retinal ganglion cells may lead to decreased visual field map sizes, and both may lead to increased pRF sizes in the cortical central visual field representation. We measured our participants’ perceptual corrected visual acuity using standard ophthalmological letter charts. We then measured their early visual field map (V1, V2 and V3) functional population receptive field (pRF) sizes and structural surface areas using fMRI, and their retinal structure using high-definition optical coherence tomography. With increasing age visual acuity decreased, pRF sizes increased, visual field maps surface areas (but not whole-brain surface areas) decreased, and retinal thickness decreased. Among these measures, only functional pRF sizes predicted perceptual visual acuity, and Bayesian statistics support a null relationship between visual acuity and cortical or retinal structure. However, pRF sizes were in turn predicted by cortical structure only (visual field map surface areas), which were only predicted by retinal structure (thickness). These results suggest that simultaneous disruptions of neural structure and function throughout the early visual system may underlie the deterioration of perceptual visual acuity in healthy aging.

**Supplementary Information:**

The online version contains supplementary material available at 10.1007/s00429-021-02338-0.

## Introduction

Low-level visual perceptual abilities, like visual acuity, decline during healthy human aging (Evans et al. 2002). Aging is associated with structural changes in the retina including a gradual loss of retinal ganglion cells and their axons (Harwerth et al. 2008; Harwerth and Wheat 2008; Pearson et al. 2006). In the early visual cortex, primary visual cortex (V1) changes in both structure (decreasing surface area) and function (increasing receptive field sizes) between young and older adults (Brewer and Barton 2012, 2014). We asked how the differences with age in the perception, structure and function of the early visual system in healthy human aging are related in a large cross-sectional sample of 50 healthy human subjects from 20 to 80 years of age. We hypothesized that age-related changes in retinal and cortical structure and function may be linked, and together may underlie perceptual deterioration.

The brain analyses visual space in a network of areas where the structural organization of the retina is repeatedly mapped onto the cortical surface (Deyoe et al. 1996; Wandell et al. 2005; Wandell and Winawer 2011). This retinotopic structural organization of early visual areas V1, V2, and V3 has been well characterized in humans (Dougherty et al. 2003; Wandell et al. 2007). Recently developed functional magnetic resonance imaging (fMRI) methods also allow the functional response selectivity of neural populations to be characterized non-invasively in vivo. This approach, population receptive field (pRF) modeling (Dumoulin and Wandell 2008), relies on the gradual progression of single-neuron receptive field positions within retinotopic visual field maps, grouping together similarly responding neurons. This is a major advance beyond localizing responsive areas and characterizing their structure: it allows comparisons of functional neural response properties between humans and even comparison of neural response selectivity to behavioral measures of perceptual abilities from the same subjects.

The visual position selectivity of neural populations (pRF size) in the early visual field maps has been well characterized in young adults. PRF sizes increase with visual eccentricity within a visual field map and increase hierarchically between visual field maps. Smaller pRF sizes imply a finer neural representation of visual space. The last few years have seen an expansion of studies linking human visual perceptual abilities to functional pRF properties. PRF sizes are smaller where visual acuity is higher, near the foveal regions of the cortex (Dumoulin and Wandell 2008; Harvey and Dumoulin 2011) and in the horizontal visual quadrants (Silva et al. 2018), reflecting the spatial resolution of visual processing. The fine-scale details of structural retinotopic organization within these visual field maps show complementary changes. The cortical magnification factor (CMF, the cortical area responding to each degree of visual angle) increases in foveal regions (Dougherty et al. 2003; Harvey and Dumoulin 2011) and horizontal quadrants (Silva et al. 2018), similarly implying a finer representation of visual space here. Recent studies in young adults have linked differences in pRF size and/or CMF to differences in perceptual performance across the visual field (Duncan and Boynton 2003; Silva et al. 2018) and between individual adults (Schwarzkopf et al. 2011; Song et al. 2015).

It has been proposed that retinal ganglion cell loss may lead to receptive field enlargement (King et al. 2006; Sharma 2008) in glaucoma models, through changes in cortical pooling mechanisms (Redmond et al. 2010) or a degradation of intracortical inhibition. We have recently used pRF properties to relate the quality of vision to neural plasticity after cataract surgery in older adults (Miranda et al. 2018; Rosa et al. 2017).

We, therefore, hypothesized that age-related loss of macular retinal ganglion cells may lead to decreased visual field map sizes, and both may lead to increased pRF sizes in the cortical central visual field representation. To test this hypothesis, we combined perceptual measures of visual acuity with two neuroimaging methods: pRF modeling of fMRI data to quantify cortical visual field map structure and function, and high-definition optical coherence tomography (OCT) to quantify retinal structure. We tested the same 50 participants in all three data sets, spanning an age range from 20 to 80 years. This allowed us to quantify age-related changes in visual acuity (perception), early visual field map receptive field sizes (cortical functional) and surface areas (cortical structure), and retinal thickness (retinal structure). It also allowed us to examine between-participant correlations in these measures to determine how the differences with age in early visual system structure, function and perception during healthy human aging are related.

## Methods

### Participants

Fifty healthy right-handed volunteers were recruited for this study and categorized by age into three groups: young adults (20–40 years, *n* = 18), middle-aged adults (40–60 years, *n* = 17), and older adults (60–80 years, *n* = 15). A full neuro-ophthalmological examination was performed, including intraocular pressure (IOP) measurement (Goldman applanation tonometer), slit-lamp biomicroscopy and fundus examination (Goldman lens) to ensure participants had no clinical disorders of the eye. Before acuity measurements and fMRI began, participants’ subjective refractive errors were measured and corrected for. After this correction, best-corrected visual acuity (BCVA) was measured as Early Treatment Diabetic Retinopathy Study (ETDRS) letter score using the ETDRS chart (higher scores correspond to better vision). Retinal image acquisition was obtained with optical coherence tomography (spectral domain Cirrus HD-OCT 5000, Carl Zeiss Meditec, Dublin, CA, USA) and only participants without any abnormalities of the macula or the optic disc were included. All participants had normal or corrected to normal vision (visual acuity ≥ 8/10) and IOP ≤ 21 mmHg, and with no history of visual disease or clinical intervention. No subject showed any signs of Age-Related Macular Degeneration (even in an early stage), nor family history of glaucoma or other hereditary eye disease or diabetes. One subject was left-handed as determined by the Edinburgh inventory (Oldfield 1971) and excluded. Only participants without cognitive impairment were included in the study as assessed using the Montreal Cognitive Assessment-MoCA, a screening tool for cognitive deterioration (Freitas et al. 2011), scoring within normality according to their age and education. None of the subjects had a history of neurological or psychiatric disorders. The study was conducted in accordance with the tenets of the Declaration of Helsinki and was approved by the Ethics Committee of the University of Coimbra. Written informed consent for the study was obtained, after an explanation of the nature and possible consequences of the study. Table 1 shows all demographic parameters of the study participants. There were no statistically significant differences between age groups in gender, education level and other demographic characteristics of participants.

**Table 1:** Participants’ demographic characteristics

Demographic parameters	Age groups		
	20–40 years	40–60 years	60–80 years
Sample size (*N* subjects)	18	17	15
Mean age (SEM) (years)	29.44 (1.15)	48.24 (1.29)	68.40 (1.51)
Age range (years)	23–38	40–56	60–79
Gender (male:female)	9:9	11:6	6:9
Mean weight (SEM) (kg)	66.11 (3.14)	73.33 (4.12)	67.04 (3.11)
Mean height (SEM) (meters)	1.68 (0.01)	1.67 (0.02)	1.67 (0.02)
Mean age of last education (SEM): range (years)	16.72 (0.61):10–20 years	15.06 (1.01): 6–20 years	13.79 (0.99): 4–17 years

### fMRI acquisition

Data acquisition was performed on a Siemens Magnetom Trio 3 T scanner (Siemens, Erlangen, Germany), using a whole-brain approach, with a 12-channel head coil. Two high-resolution 3D anatomical MPRAGE (magnetization-​prepared rapid gradient-echo) T1-weighted sequences were acquired, each with an isotropic resolution of 1 mm, repetition time (TR) of 2530 ms, echo time (TE) of 3.42 ms, field of view (FOV) of 256 × 256 mm. Each anatomical sequence comprised 176 slices, a flip angle of 7°, an inversion time of 1100 ms with a total time of 363 ms. The functional T2*-weighted 2D echo-planar MRI images were acquired with an isotropic resolution of 2 mm, TR of 2000 ms, TE of 30 ms, FOV of 256 × 256 mm. Each functional sequence comprised 29 interleaved slices and 186 volumes, the first six initial volumes for BOLD stabilization were discarded.

### Visual stimuli

During fMRI visual field mapping, participants wore any corrective lenses assigned during the initial ophthalmological examination. Visual stimuli were displayed on a 32-inch LCD monitor (Inroom Viewing Device; NordicNeuroLab, Bergen, Norway) with 1920 × 1080 pixel resolution, positioned at the end of the scanner bore and viewed through a mirror attached to a head coil. The display was 70.0 × 39.5 cm and the viewing distance was 156.5 cm, so it subtended a 25.2° × 14.4° of visual angle.

The visual field mapping stimulus was created with PsychToolbox (Brainard 1997; Pelli 1997) for Matlab (version R2014b; Mathworks, Natick, MA, USA). It consisted of bars stepping perpendicular to bar orientation across a 7.2° radius circle filling the display’s vertical dimension (Dumoulin and Wandell 2008). These bars contained a white and black checkerboard pattern with 100% contrast moving parallel to the bar orientation (vertical, horizontal, and diagonal). The bar was 1.80° wide, 1/4th of the stimulus radius, and the checks were each 0.9° square, so the spatial frequency of the checkerboard matched the bar width. Four bar orientations (0°, 45°, 90°, and 135°) and two different motion directions for each bar were presented giving a total of eight different bar motion directions, each of which crossed the display in 15 steps of 1.03°, lasting 2 s each, 30 s total. Four 30 s mean luminance (0% contrast) blocks were presented, one after each horizontal or vertical oriented bar crossing. Participants completed four visual field mapping runs (240 time frames each, 6 min) within the same session.

A fixation dot at the center of the visual stimulus changed from red to green at random time intervals and participants were instructed to press a button on a response box every time they detected a color change, to ensure that attention and fixation were maintained. Color changes were every 3 s on average, with a minimum change interval of 1.8 s. We discarded any scan where detection performance dropped below 70% (2 scans of 1 subject). Mann–Whitney comparisons on the number of correct responses revealed no significant difference in performance between age groups (*U* = 28.000, *p* = 0.112).

### Anatomical and functional preprocessing

All fMRI data were processed and analyzed using BrainVoyager QX software (v2.8.4; Brain Innovation, Maastricht, the Netherlands). First, anatomical data underwent brain extraction and intensity inhomogeneity correction to reduce artifacts and inhomogeneity caused by the magnetic field (Dale et al. 1999). The two anatomic data sets were aligned to each other and to improve the signal-to-noise ratio, were averaged and re-oriented into AC–PC plane, followed by transformation to the Talairach reference system. The white matter was segmented using an automatic segmentation routine (Kriegeskorte and Goebel 2001) and small manual adjustments were made, to create surface representations of each hemisphere (meshes). Preprocessing of the functional data included slice time correction, linear trend removal, temporal high-pass filtering (up to 2 cycles per scan), and 3D motion correction (rigid body) with spline interpolation. All volumes were corrected for head movement and motion artifacts between and within functional scans. Then, they were coregistered with each subject’s structural scan in Tailarach space and averaged across scans (Nestares and Heeger 2000).

### Population receptive field estimation and analysis

PRF models were estimated from the fMRI data and the time course of visual stimulus positions using a model-driven approach (Dumoulin and Wandell 2008) and implemented in BrainVoyager QX. Briefly, this approach estimates a neural response model, for each voxel, that best explains each voxel’s fMRI response to the stimulus’s visual field positions. It models the preferred position (*x* and *y*) and size (standard deviation or σ) of a two-dimensional circular Gaussian function describing the area of the visual field to which the voxel responds. First of all, we generated a binary stimulus aperture containing the visual field positions covered by the stimulus bars in each TR. Next, for a large set of combinations of pRF positions and sizes, we calculated the proportion of the pRF Gaussian overlapping the stimulus aperture at every TR, proportional to the predicted neural response amplitude for this candidate pRF and this stimulus.

Next, each candidate neural response time course was convolved with a canonical BOLD hemodynamic response function to predict the BOLD response time course that the stimulus would yield for this set of pRF parameters. For each voxel, the pRF parameters (preferred position and size) were found that predict the BOLD response time course best correlated to the voxel’s response time course, with the variance explained by the model being equivalent to the *R*^2^ of this correlation.

The preferred position for each voxel was converted to preferred eccentricity and polar angle. These resulting parameter maps were projected onto inflated cortical meshes (Wandell et al. 2000), and the positions of V1, V2 and V3 were defined as regions of interest (ROIs) in relation to visual field representations (Fig. 2) (Dougherty et al. 2003; Sereno et al. 1995; Wandell et al. 2007) using BrainVoyager’s surface drawing tools. The surface area of these ROIs was determined at the grey-white matter boundary, to avoid the effects of grey matter thinning. To test for effects that are not limited to the visual cortex, the surface area of each hemisphere with these ROIs removed was also determined. Voxels with pRF model variance explained below 0.3 were excluded from further analysis, as were voxels outside of the delineated ROI. We also excluded voxels with pRF eccentricities below 0.5°, since this part of the visual field is difficult to accurately map, and those beyond 6° eccentricity (near the edge of our stimulus area, where pRF properties are not estimated reliably. We attempted to delineate higher order areas, such as V4 and V3A, but in many participants, these could not be identified reliably, because model fits were poor in those instances.

### Retinal imaging/thickness acquisition

Measurements of macular retinal thickness (RT), retinal nerve fiber layer thickness (RNFLT) and the ganglion cell-inner plexiform layer thickness (GCIPLT) were obtained with a Cirrus HD-OCT 5000 (Zeiss Meditec, Jena, Germany). All acquisitions were performed by the same trained operator. The volumetric data with 512 × 128 × 1024 voxels were acquired using the Macular Cube protocol centered on the macula. This protocol generates a cube of data through a 6 mm square grid around the fovea centralis by acquiring a series of 128 horizontal B-scans lines each composed of 512 A-scans, with an axial resolution of 5 mm. The standard output display of Cirrus HD-OCT includes a color topographical and a thickness map displaying measurements calculated for each of the nine macular areas corresponding to and defined by ETDRS grid. The global macular RT is defined as the average macular thickness from the inner limiting membrane to the top of the retinal pigment epithelium over the entire 6 × 6 scanned area. Global RT (mean of thicknesses in the nine sections of the ETDRS grid) was determined automatically and analyzed by the Cirrus HD-OCT’s internal algorithm. The thickness of two retinal layers was determined as well, RNFLT and GCIPLT. The RNFLT was acquired using the optic disc cube 200 × 200 protocol and analysis uses a 3.46 mm circle centered and around the optic disc. The ganglion cell analysis (GCA) algorithm measured the macular GCIPLT, within a 14.13 mm^2^ elliptical annulus area centered on the fovea. The global GCIPLT was measured in an elliptical annulus of the macular cube scan mode. Both eyes of each participant were separately scanned and compared, and no statistically significant differences between right and left eyes were found.

### Statistical analysis

A MATLAB (version R2019b, Statistics and Machine Learning Toolbox) script was developed to extract the mean pRF size of each visual area for each participant (see Supplementary Materials). We then tested the effect of age on acuity (BCVA), visual field map pRF sizes, visual field map surface areas and retinal thickness measures using Spearman (non-parametric, rank) correlations. For consistency, we used non-parametric statistics throughout as some measures significantly deviated from normal distributions (in Shapiro–Wilk tests). We then tested for relationships between these measures, again using Spearman correlations. Results with *p* < 0.05 were considered statistically significant.

We also used Bayesian Kendall’s tau analysis (van Doorn et al. 2018) to quantify support for or against the null hypothesis of no relationships between the ranks of these measures. We use van Doorn and colleagues’ method for determining priors in this test (implemented in JASP), where the null hypothesis prior is centered at 0 and its spread is estimated from the data.

We also tested a set of general linear models of retinal thickness, V1 surface area, V1 global pRF size, and visual acuity, where each of these was used as a dependent variable, and the remaining three as independent predictors acting together.

Finally, we used a non-parametric bootstrapped mediation analysis (Preacher and Hayes 2008) to ask whether the major relationships we observe have significant components that are independent of age.

Where two measurements are possible from each subject, either from the two eyes (retinal thickness) or the two hemispheres (pRF size, visual field map surface area), we treat these as two independent measurements. However, where testing for a correlation (or GLM) including a two-eye measure and a two-hemisphere measure, these pairs do not match up. Here we use the mean retinal thickness from both eyes, the mean pRF size from both hemispheres, or the summed surface area from both hemispheres.

## Results

### Visual acuity decreased with age

**Fig. 1:**
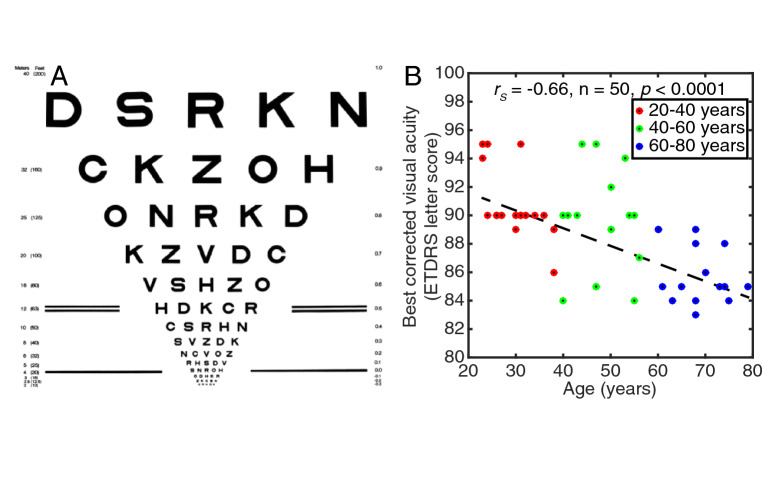
Visual acuity decreased with age. **A** Example test card for measuring best-corrected visual acuity, from the National Eye Institute, National Institutes of Health. **B** Acuity decreased with age. Points are individual participants, dashed line is best linear fit

We quantified best-corrected visual acuity (BCVA) using binocular ETDRS letter score (Fig. 1A), where higher values indicate better vision. BCVA was significantly negatively correlated with age, with letter score decreasing by 1.25 points per decade (Fig. 1B, statistics shown on figures).

### Visual field map pRF sizes and surface areas changed with age, and were correlated, but only pRF sizes were correlated with acuity

**Fig. 2:**
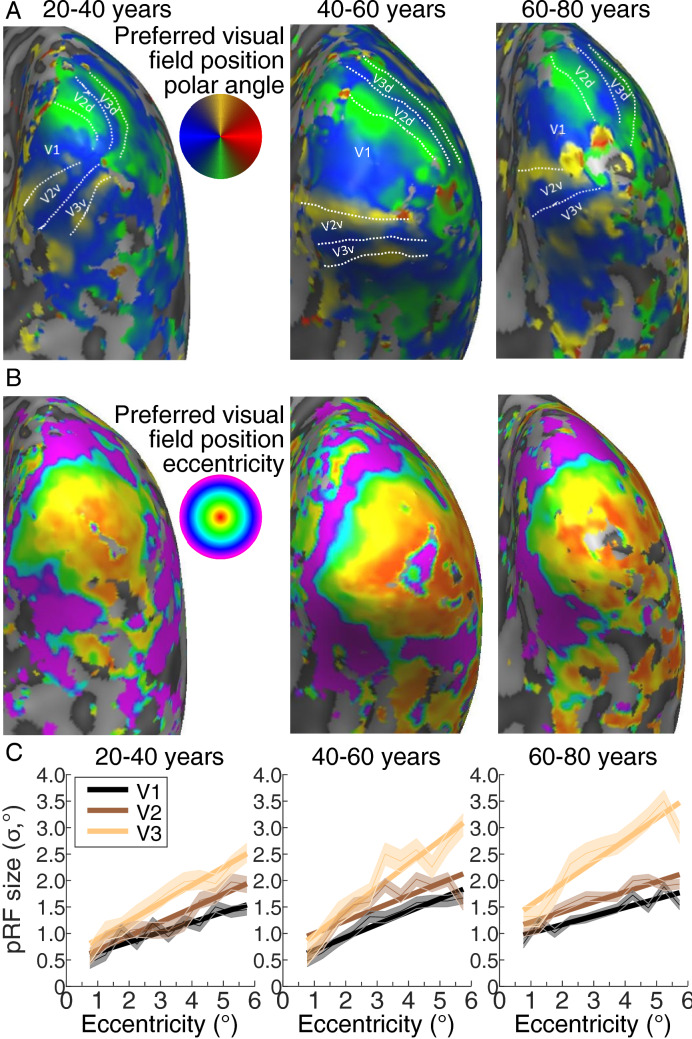
Visual field maps and pRF size changes with eccentricity across the early visual cortex. **A** Polar angle maps displayed on the inflated mesh (right hemisphere) for a representative example of each age group. The colors represent the recording sites for which the pRF model explains at least 30% of the variance. White dotted lines and labels show the position of the identified visual field maps. **B** Eccentricity maps displayed on the right hemisphere inflated mesh of the same representative participant of each age group. **C** PRF size changes with eccentricity in V1, V2 and V3 for the same participant from each age group. Shaded areas show the mean ± 1 SEM within each 0.5° eccentricity bin. Solid lines represent the best linear fit to bin means

Maps of preferred visual field position polar angle and eccentricity across the early visual cortex were taken from pRF models for each participant. Figure 2A, B shows a representative hemisphere from young (20–40 years), middle-aged (40–60 years) and older (60–80 years) adults. All ages showed normal organization in these visual field maps. The visual field maps (V1, V2 and V3) were manually delineated for each participant.

**Fig. 3:**
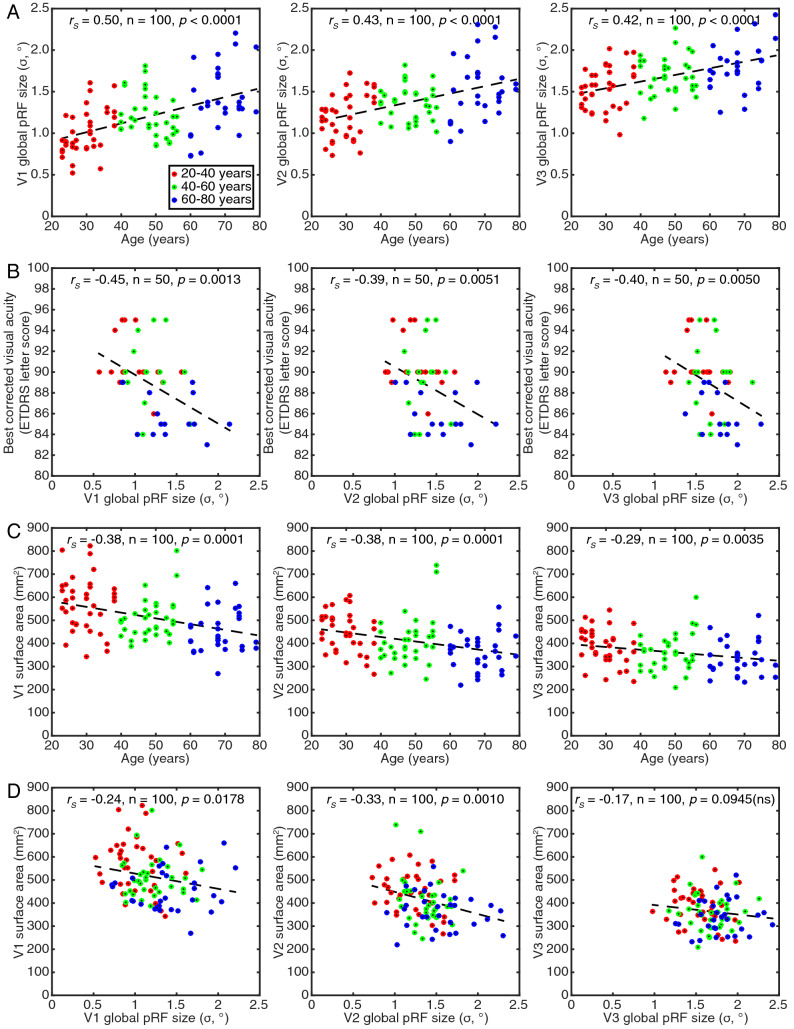
Age-related changes in global pRF size, surface area and their relationships. **A** Global pRF size increased with age. **B** BCVA decreased with increasing global pRF size. **C** Visual field map surface area decreased with age. **D** Visual field map surface area decreased as global pRF size increases in V1 and V2. Points are individual hemispheres (individual subjects in **B**), dashed line is the best linear fit

We then binned the pRF size of the recording sites in each visual field map into eleven eccentricity bins, each 0.5° wide, from 0.5° to 6°. Figure 2C shows changes in pRF size with eccentricity in V1, V2 and V3 representative example participants from each age group. To summarize these for each participant, we took a mean of these bins (global pRF size). We did not use the mean of individual recording site pRFs to avoid eccentricity-related differences in cortical magnification factors affecting these means. Low eccentricities contribute more to mean pRF sizes than high eccentricities because cortical magnification decreases with eccentricity. Furthermore, age-related differences in the distribution of cortical magnification factors would affect mean pRF size measures. Global pRF size avoids these issues, giving a summary pRF size measure that is independent of cortical magnification factor. Global pRF sizes increased with age, showing strong correlations with age in V1, V2 and V3 (Fig. 3A). Global pRF sizes increased across age in V1 by 0.11° per decade (i.e., 8.8% of the average hemisphere’s global pRF size), in V2 by 0.09° (i.e., 6.5%) and in V3 by 0.08° (i.e., 4.7%). Global pRF sizes were strongly negatively correlated with visual acuity (Fig. 3B), as previously shown within a narrower age range (Song et al. 2015). A Bayesian Kendall’s tau analysis (van Doorn et al. 2018) also supports the alternative hypothesis of a relationship between pRF size and visual acuity in all of these visual field maps (V1: BF_10_ = 19.3. V2: BF_10_ = 3.6. V1: BF_10_ = 6.9).

Next, we asked whether global pRF sizes in different visual field maps were correlated across subjects. While not specifically relevant for questions of age and acuity, we can find no previous test of this relationship. We found strongly significantly correlations between pRF sizes in all visual field map pairs (V1 and V2: *r* = 0.797, *p* < 0.0001; V2 and V3: *r* = 0.708, *p* < 0.0001; V1 and V3: *r* = 0.583, *p* < 0.0001; all *n* = 100).

To summarize each participant’s visual field map size, we measured the total surface area (in mm^2^) covered in each map from 0.5° to 6° eccentricity. Visual field map surface areas decreased with age, showing strong negative correlations in V1, V2 and V3 (Fig. 3C). Each hemisphere’s V1 decreased across age by 25 mm^2^ per decade (i.e., 4.8% of the average hemisphere’s surface area), V2 by 19 mm^2^ (i.e., 4.6%), and V3 by 12 mm^2^ (i.e., 3.3%). So, age-related changes in both the visual field maps pRF sizes and surface area appear to decrease up the hierarchy. Indeed, the surface areas of the rest of the hemisphere (excluding V1, V2 and V3) were not significantly correlated with age (*r* = − 0.13, *p* = 0.19, *n* = 100). The surface areas of V1 and V2 were significantly negatively correlated with their pRF sizes (Fig. 3D), while the correlation of V3’s surface area and pRF size did not reach significance on a two-sided test (*p* = 0.094). This correlation has been shown before in V1, within a narrower age range (Harvey and Dumoulin 2011). However, no visual field map showed any significant relationship between its surface area and visual acuity (V1: *p* = 0.084; V2: *p* = 0.285; V3: *p* = 0.790). Therefore, visual acuity followed pRF size but not surface areas of early visual field maps. In contrast to the relationship between pRF sizes and acuity, a Bayesian Kendall’s tau analysis found no evidence for or against a null hypothesis of no correlation between V1 surface area and visual acuity (BF_01_ = 1.0), and supports the null hypothesis of no correlation between V2 and V3 surface area and visual acuity (V2: BF_01_ = 3.1. V3: BF_01_ = 5.3).

### Retinal thickness decreased with age and predicted visual field map surface areas

**Fig. 4:**
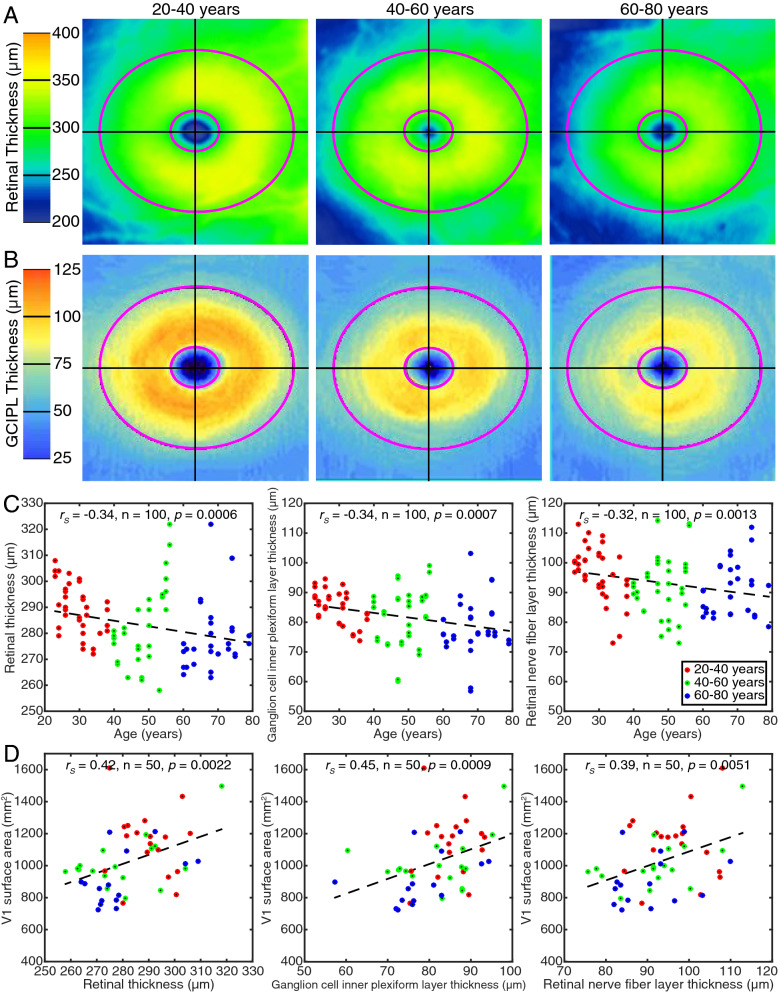
Age-related retinal thinning predicted decreases in V1 surface area. **A** Global retinal thickness maps for foveal region of the right eyes of representative participants from each age group. The inner ellipse (0.6 mm horizontal by 0.5 mm vertical diameter, or approx. 2° by 1° 40′) covers the foveola, while the outer ellipse (2.4 mm by 2.0 mm, or approx. 8° by 6° 40′) covers the fovea. Retinal thickness measures are averaged within this region. **B** Corresponding ganglion cell inner plexiform layer thickness maps. **C** The thickness of the total retina, ganglion cell inner plexiform layer and retinal nerve fiber layer decreased with age. **D** All retinal thickness measures predicted V1 surface area, but not pRF size or acuity. Points are individual eyes (in **C**) or participants (in **D**) dashed line is the best linear fit

**Fig. 5:**
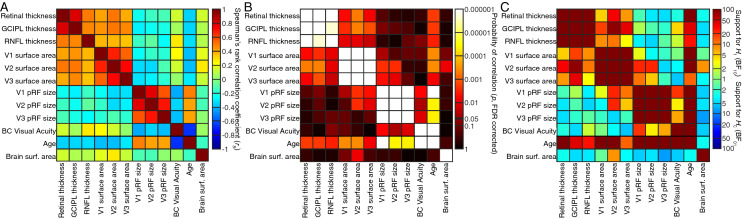
Correlations between all measures taken. **A** Pairwise Spearman’s correlation coefficients for all pairs of measures taken. **B** Probability of these correlations, after false discovery rate (FDR) correction for multiple comparisons. **C** Support for null and alternative hypotheses of correlations in Bayesian Kendall’s tau analyses

We measured global mean RT (Fig. 4A), GCIPLT (Fig. 4B), and RNFLT in all participants. The measured values were consistent with normative data of Liu et al. 2001. All three measures were significantly negatively correlated with age (Fig. 4C), with RT decreasing across age by 2.2 µm, GCIPLT decreasing 1.5 µm and RNFLT decreasing 1.5 µm per decade. All three retinal measures were significantly correlated with the surface area of V1 (Fig. 4D) and also V2 and V3 (Fig. 5). However, no retinal measure was significantly correlated with pRF size (in any visual field map) or with visual acuity. Bayesian Kendall’s tau analyses supported the hypothesis of a correlation between V1 surface area and all retinal thickness measures (RT and V1 surface area: BF_10_ = 4.7. GCIPLT and V1 surface area: BF_10_ = 17.7. RNFLT and V1 surface area: BF_10_ = 8.4) but supported the null hypothesis of no correlation between retinal thickness and both pRF size (RT and V1 pRF size: BF_01_ = 1.7. RT and V2 pRF size: BF_01_ = 3.8. RT and V3 pRF size: BF_01_ = 4.2) and visual acuity (BF_01_ = 2.4).

### Integrated comparisons

The overall pattern of results so far suggests that retinal thickness predicted visual field map surface areas, surface areas predicted pRF sizes, and pRF sizes predicted visual acuity. This is particularly evident in Fig. 5B, where p values of correlations become less significant (darker) with distance from the diagonal (i.e. with steps from the retina to perception). Indeed, correlations no longer reached significance when measures are separated more than one of these steps and Bayesian statistics support the null hypothesis of no relationship here.

However, we have tested this using separate correlations, and some of these measures are closely related and co-vary. Therefore, we also tested a set of general linear models of retinal thickness, V1 surface area, V1 global pRF size, and visual acuity, where each of these was used as a dependent variable, and the remaining three as independent predictors acting together. This demonstrated that V1 global pRF size and visual acuity significantly predicted each other (*t* = − 3.45, d*f* = 46, *p* = 0.0012), while other measures did not significantly predict either. Similarly, retinal thickness and V1 surface area significantly predicted each other (*t* = 2.264, d*f* = 46, *p* = 0.0054), while other measures did not significantly predict either. V1 surface area and pRF size did not predict each other here, apparently because the variance in these measures was better captured by retinal thickness and visual acuity respectively, to which they were more strongly correlated. Nevertheless, significant correlations between V1 surface area and pRF size have been previously demonstrated (Harvey and Dumoulin 2011), and were replicated here (Fig. 3D).

Finally, investigating how different measures contribute to each other is complicated by the fact that all of our measures are strongly predicted by a common factor that seems likely to mediate these relationships: age. We used a non-parametric bootstrapped mediation analysis (Preacher and Hayes 2008) to ask whether the major relationships we observed had significant components that were independent of age. First, we asked whether the effect of age on visual acuity had a separable component that was mediated by V1 global pRF size, as all of these measures were correlated with each other. In this model, the effect of age on visual acuity (*t* = 2.78, *p* = 0.0077, d*f* = 49) consisted of a significant direct component (*t* = 2.14, *p* = 0.037 d*f* = 48), but the component mediated by V1 pRF size did not reach significance (*t* = 0.34, *p* = 0.74, d*f* = 48). Next, we asked whether the effect of age on V1 global pRF size had a separable component that was mediated by V1 surface area. Again, the effect of age on V1 global pRF size (*t* = 4.37, *p* = 0.00006, d*f* = 49) consisted of a significant direct component (*t* = 3.98, *p* = 0.0002 d*f* = 48), but the component mediated by V1 surface area did not reach significance (*t* = 0.11, *p* = 0.91, d*f* = 48). Finally, we asked whether the effect of age on V1 surface had a separable component that was mediated by retinal thickness. Again, the effect of age on V1 surface area (*t* = 3.20, *p* = 0.0024, d*f* = 49) consisted of a significant direct component (*t* = 2.62, *p* = 0.0119, d*f* = 48), but the component mediated by retinal thickness did not reach significance (*t* = 1.39, *p* = 0.17, d*f* = 48).

## Discussion

In this study, we examined the neural basis of the common decline in visual acuity during healthy human aging by measuring visual acuity, retinal thickness, early visual field map surface areas, and their population receptive field (pRF) sizes in 50 adults from 20 to 80 years old. We characterized how these measures changed with age and how they co-varied. Retinal thickness, visual field map surface areas, and visual acuity all decreased with age, while pRF sizes increased. All these changes imply coarser visual processing. However, among these measures of neural structure and function, only functional pRF size significantly predicted visual acuity. Indeed, Bayesian statistics supported the null hypothesis that retinal thickness measures and visual field map surface areas were unrelated to visual acuity. PRF size was in turn predicted only by the visual field map’s surface area, which was in turn predicted by retinal thickness measures. However, it is important to note that these relationships were derived by correlations, and all of these changes were strongly correlated with age, a common factor affecting all measures. We did not find significant mediation of changes in acuity by changes in pRF size, pRF size by visual field map surface area, or visual field map surface area by retinal thickness that was separable from effects of age.

Our pRF size results are consistent with previously described increases in pRF size up the visual hierarchy, with recording sites’ preferred eccentricities, and in subjects with smaller V1 surface areas (Harvey and Dumoulin 2011). Previous results have also shown smaller pRF sizes in V1 and V2 predict lower perceptual position discrimination thresholds (Song et al. 2015). These results all come from young and early middle-aged adults, 19–47 years old. Previous studies comparing 5 healthy young adults (24–36 years) and 4 healthy older adults (57–70 years) have shown larger pRF sizes and smaller V1 surface areas in the older group (Brewer and Barton 2012, 2014). These two studies use the same data, from very small numbers of participants. This small sample size is concerning because pRF sizes and visual field map surface areas vary by a factor of two to three between healthy young adults (Dougherty et al. 2003; Harvey and Dumoulin 2011). We confirm this difference in our larger sample, and also show that pRF sizes are beginning to increase (and acuity decrease) in middle age. This suggests that age may be an important factor in individual differences in pRF size and acuity, and their covariation, even among the common sample of young and early middle-aged participants (Song et al. 2015). However, the pRF size differences seen between young and middle-aged groups (an increase of ~ 20%) are insufficient to explain the full range of individual differences (~ 250%).

Regarding the retina, our OCT measures of retinal structure are also consistent with previous reports of gradual loss of retinal ganglion cells and their axons during healthy aging (Harwerth et al. 2008; Harwerth and Wheat 2008; Jorge et al. 2020; Pearson et al. 2006). We propose that this retinal ganglion cell loss is likely to cause the decreases in visual field map surface areas, and (indirectly) the increases in pRF size, that we observe. For changes in visual field map surface areas, degradation of ascending retinal ganglion cell projections (in macular degeneration) causes a decrease in the gray matter volume in the affected cortical projection zone (Hanson et al. 2019). Although V2 and V3 do not receive direct ascending projections, V2 is physically linked to V1 and V2 size is correlated with V1 size (Dougherty et al. 2003). Also, degradation of V1 may cause a similar atrophy of V2 and V3 through a reduction in feedforward activity. Interestingly, the decrease of surface area across age in V1 appears to occur faster (25 mm^2^ per decade, or 4.8%) than in V2 (19 mm^2^ per decade, or 4.6%), which is in turn faster than in V3 (12 mm^2^ per decade, or 3.3%). This may suggest an effect in V1 being passed up the hierarchy.

Increases in early visual receptive field and pRF sizes are closely coupled to decreases in visual field map surface area or cortical magnification factor between individuals and within visual field maps (Harvey and Dumoulin 2011; Hubel and Wiesel 1974; Silva et al. 2018). Here we measure pRF size, the aggregate receptive field size of the neural population within an fMRI voxel. Is it possible that the observed increase in pRF sizes simply reflects the decrease of the visual field maps’ surface area, such that more visual space falls into one voxel’s visual field map extent? We believe this is unlikely. First, the observed decrease of visual field map surface areas across age (4.8% per decade in V1) is far less than the observed increase of pRF size (8.8% per decade in V1) so seems unlikely to fully account for pRF expansion. Second, the spatial extent of the visual field map sampled within a voxel seems to have little effect on pRF size, as neither voxel size nor fMRI field strength (which affects fMRI’s spatial specificity) affects pRF size estimates (Haak et al. 2013; Morgan and Schwarzkopf 2020). Finally, in our mediation analysis, the component mediated by V1 surface area does not reach significance in predicting V1 pRF sizes. Therefore, while there are close relationships between pRF size and local cortical magnification, these relationships are also found for single-neuron receptive field sizes and seem to be primarily biological rather than methodological in origin.

In averaging a visual field map’s pRF sizes we aim to avoid biases that would result from potential age-related changes in cortical magnification distributions. We, therefore, bin our data by eccentricity then average these bins, so that eccentricity ranges containing more voxels do not contribute more to global mean pRF size estimates. However, eccentricity is not linearly distributed within a visual field map: more voxels have low than high eccentricities. This binning therefore still includes a smaller bias, because most voxels in an eccentricity bin have eccentricities and pRF sizes below the bin mean. The resulting underestimation of pRF sizes would be affected by potential age-related changes in cortical magnification distributions, and also potentially by age-related changes in the variability in pRF size estimates. However, if the variability of pRF size estimates or the slope of the cortical magnification function increased across age (for example as a result of measuring in a smaller visual field map) these effects seem most likely to underestimate global pRF sizes, while we find greater global pRF sizes in older subjects.

Retinal ganglion cell loss is also likely to indirectly affect receptive field sizes through cortical mechanisms: changes in cortical pooling (Redmond et al. 2010) or a degradation of intracortical inhibition, as seen in glaucoma models (King et al. 2006; Sharma 2008). Previous results from senescent primates show a decrease (broadening) of orientation and motion direction selectivity in V1 and V2, coupled with increases in neural excitability (Leventhal et al. 2003; Schmolesky et al. 2000; Yu et al. 2006). These findings suggest an age-related degradation of intracortical inhibition resulting from the reliance of extrastriate receptive field properties on the upstream V1 receptive fields. As pRF sizes in extrastriate visual cortex (V2 and V3) are correlated with V1 pRF sizes, this increase in pRF size is likely to cascade through the visual hierarchy. Indeed, we see age-related increases in pRF size at least up to V3, with the change in pRF sizes again decreasing from V1 (0.11° or 8.8% per decade) to V2 (0.09° or 6.5%) to V3 (0.08° or 4.7%) even as the pRF sizes themselves increase. Given that large pRF sizes in early visual field maps predict high visual position discrimination thresholds (Song et al. 2015), such changes may underlie age-related reductions in visual acuity, though this relationship is complicated by changes in all measurements with age.

We found that only pRF sizes predicted acuity, that pRF sizes were in turn predicted by visual field map surface areas, and that surface areas are in turn predicted by retinal thickness. However, it is too soon to make mechanistic conclusions about the causation of acuity deterioration from this simultaneous set of changes. First, our mediation analyses show that all of these measures follow age, but no measure significantly mediates the effects of age on any other. Second, measurements of pRF size (and indeed any measurement of receptive field size) depend on several neural and non-neural factors. As some of the non-neural factors change with age, it is not possible to unequivocally attribute pRF size increases to a broadening of neural response functions. For example, pRF size estimates may be affected by age-related deterioration of the eye’s optical properties. During pRF mapping and acuity measurements our participants wore any corrective lenses assigned to them in the initial ophthalmological examination, but these correct refractive errors only and may not do so perfectly. While participants had no cataracts, it is hard to exclude the possibility of imperfect optics. Age-related changes in the size or rigidity of the pupils may also affect the optical properties of the eye. Similarly, older participants may show a slight instability of fixation that would increase pRF size estimates and may also affect acuity. Notably, deterioration of the eyes’ optical properties, fixation stability and pupil responsivity could be expected to reduce visual acuity as well as increasing pRF size. So decreases in acuity and increases in pRF size may have a third cause, rather than increases in pRF size causing decreases in acuity. Speaking against this interpretation, V1 pRF sizes increase from around 1° (standard deviation) in the average 30-year-old to around 1.5° in the average 75-year-old, which would require convolution with an optical blur or a spread of gaze positions with a 1.12° standard deviation to explain. That much blurring would severely disrupt vision and seems unlikely among participants with normal vision, and that much spread of gaze position is unfeasibly large. Furthermore, while 60–80-year-old participants may well show some optical blur and fixation instability, pRF sizes are already increasing in our 40–60-year-old participants, where extensive optical blur and fixation instability are unlikely. But it is nevertheless important to remember that optical imperfections and fixation instability would affect acuity and pRF properties but not visual field map surface area or retinal thickness.

It is also important to remember that pRF properties are estimated from fMRI BOLD responses. BOLD responses reflect a change in blood flow: hemodynamic responses following neural activity. It may be that age-related effects on pRF size reflect (at least in part) age-related changes in the hemodynamic response: a spatial broadening or temporal slowing of hemodynamic responses would both predict increased pRF size estimates. Hemodynamic changes alone seem unlikely to account for all changes in pRF properties because pRF size changes are correlated with changes in both perceptual acuity and visual field map surface areas, which would not be affected by hemodynamic changes. Furthermore, the time course of the hemodynamic response function is not strongly affected by age (Gauthier et al. 2013). On the other hand, a hemodynamic response with the same cortical extent in a smaller visual field map would integrate responses covering a larger extent of visual space, so the decrease of visual field map surface areas across age itself may (at least in part) affect pRF sizes through hemodynamic mechanisms. An increase in participants’ head motion with age might also increase pRF size estimates, but we found no increase in head motion with age.

To measure the quality of our participants’ vision, we use best-corrected visual acuity, which measures participants' ability to read a chart of differently sized letters while wearing the best possible corrective lenses. This measure of visual acuity is widely used in ophthalmological practice and in our previous research on the quality of vision (Miranda et al. 2018). It is particularly straightforward to measure as participants are familiar with this simple test, and it requires no training, complex equipment or complex instructions. However, this test only measures acuity at fixation, rather than measuring systematically throughout the visual field (like our fMRI and OCT measures do). It primarily measures acuity, which has previously been shown to be related to pRF size (Song et al. 2015), but is likely to be less accurate than more careful and complex tests that are widespread in perceptual psychology and have been used elsewhere.

Together, our findings provide an integrated account of changes in perceptual visual acuity, retinal structure, and the structural organization and functional response selectivity of the early visual cortex during healthy aging. All of these measures were closely correlated with age, but not all were closely correlated with each other. One interpretation of this pattern is that deterioration of ascending retinal ganglion cells during healthy aging leads to a specific shrinkage of the cortical target of the ascending visual pathway, the primary visual cortex. This in turn disrupts cortical neural interactions that normally sharpen visual position selectivity, leading to an increase in cortical receptive field sizes that cascades through the early visual hierarchy. If so, these changes in functional neural response selectivity are ultimately responsible for the age-related deterioration of visual perception, but themselves follow retinal deterioration. Therapies targeting the deterioration of the retinal ganglion cells may therefore prevent all these changes, so may be a promising approach to minimize the deterioration of visual perception during healthy aging.

## Supplementary Information

Below is the link to the electronic supplementary material.

Supplementary file1 (XLSX 20 KB)

Supplementary file2 (XLSX 14 KB)

Supplementary file3 (M 11 KB)

## Data Availability

The preprocessed data on which our analyses are based are included as Supplementary material. Raw data are available on request from the corresponding author.

## Code availability

Custom code is included as Supplementary material.
